# Evaluation of dynamic F wave parameters before and after physical activity in normal population

**DOI:** 10.1186/s13104-022-06181-2

**Published:** 2022-09-09

**Authors:** Saeid Khosrawi, Shila Haghighat, Hadi Hamedfar

**Affiliations:** Department of Physical Medicine and Rehabilitation, School of Medicine, Isfahan University of Medical Sciences, Isfahan, Iran

**Keywords:** F Wave, Dynamic F wave, Physical activity, Normal population, Lower limb, Nerve conduction study

## Abstract

**Objectives:**

F wave is one of the nerve conduction studies, mainly used to study the proximal pathway of peripheral nerves. One of its applications is dynamic assessment, i.e., before and after physical activity, when symptoms develop in some patients. Best to our knowledge, few studies evaluate normal dynamic F wave values in healthy individuals. This study aims to determine and compare normal values of several parameters of Compound Muscle Action Potential (CMAP) and F waves of tibial nerves dynamically before and after physical activity in the lower extremities of 34 normal subjects. These parameters were recorded before and after 15 min of continuous walking at intervals of 1, 5, and 10 min after the end of physical activity.

**Results:**

Normal values of CMAP onset latency and amplitude, minimum, maximum, and mean latency of 10 F waves, F chronodispersion, and F persistence were dynamically collected before and during 10 min after physical activity in three phases. Some of the parameters showed significant changes (P < 0.05). Although physical activity showed statistically significant effects on some CMAP and F wave parameters (especially F chronodispersion) in normal subjects, none of them exceeded the normal clinical values introduced in the literature.

## Introduction

F wave is a delayed motor response obtained after a compound muscle action potential (CMAP). By stimulating a peripheral nerve, the excitation path to the distal elicits a CMAP response from the corresponding muscle. The same stimulation also rises in the proximal motor pathway to alpha motor neurons in the anterior horn of the spinal cord. Then, a small number of anterior horn neurons fire during the backfiring mechanism that potentially travels orthodromically toward the distal, which is recorded by the corresponding muscle called the F wave. In successive excitations, F waves are recorded with variable shapes and parameters [1, 2].

F wave travels in both the afferent and efferent pathways in the motor nerve, so damage to any part of the afferent or efferent pathway may be manifested by changes in F response parameters [1, 2]. Major diseases that the F wave helps to diagnose are of two categories. The first category contains the diseases that involve long distances of nerves, such as peripheral neuropathy due to diabetes [3, 4] or multifocal motor neuropathy [5]. The second category contains the diseases involving the proximal part of the nerve pathway while the distal part is healthy, such as radiculopathies [6] and plexopathies [7].

One of the uses of the F wave is to evaluate this wave in different situations and attempt to induce symptoms in some patients [8]. One of these methods is to record the F wave dynamically, i.e., before and after physical activity, and to create clinical symptoms in patients with several pathologies such as neurogenic claudication due to spinal canal stenosis [9, 10], lumbar radiculopathy [11] and vascular claudication due to peripheral arterial occlusive disease [12].

However, despite the above studies and the role of dynamic F wave evaluation in diagnosing peripheral neurovascular diseases, few studies have evaluated parameters of F wave dynamically in the normal and healthy populations. Manganotti et al. study examined dynamic F wave of the tibial nerve in 22 patients and the peroneal nerve in 16 patients [13]. Some other studies evaluated dynamic F wave as the control group in their study [11, 12]. This is important because normal and reference values are prerequisite to use different tests in patients and determine whether the result is normal or not. In addition, researching to obtain normal and reference parameters for specific populations is one of the common studies in nerve conduction study (NCS) [14, 15]. In the case of a routine F wave study, normal parameters for the Iranian population have been evaluated [16]. Still, normal values of dynamic F wave have not been evaluated dynamically and compared before and after physical activity.

Therefore, we decided to determine and compare F wave parameters dynamically before and 1, 5, and 10 min after physical activity in normal and healthy subjects in this study.

## Main text

### Study population

Thirty-four healthy subjects were included in this study, of which 23 (67.6%) were male, and 11 (32.4%) were female. The mean ± standard deviation of subjects' age was 31.79 ± 7.23 years old.

The subjects were enrolled in the study due to certain criteria. The inclusion criteria were as follows: Men and women aged from 18 to 65 with normal general and neurological history and physical examinations, without history of any neurologic diseases (such as spinal canal stenosis, radiculopathies, etc.) and any underlying medical diseases such as diabetes, hypothyroidism, etc. Any disturbances in the examination of lower extremities, such as decreased muscle strength, reduction or absence of deep tendon reflexes, or sensory disturbances, even if the person did not have a known disease, prevented him/her from entering the study.

Exclusion criteria were intolerance of activity, premature fatigue, and neurological symptoms following activity, and the unwillingness of the patient to continue the study.

The sample size was calculated based on the formula: n = ((z1 + z2) s ^ 2)/d ^ 2 and 95% confidence interval (Z1 = 1.96) and test power factor 80% (Z2 = 0.84). The ratio of minimum means the difference of each variable that shows a significant difference (d) to the standard deviation of the change of each variable (s) equals half (d = 0.5 s), which resulted the number of at least 32 subjects. Sampling was done by convenience method.

### Study procedure

This prospective semi-experimental single-group study was performed in neuromuscular clinics affiliated with the Department of Physical Medicine and Rehabilitation of Isfahan University of Medical Sciences in 2021.

At first, the study procedure was explained to the individuals, and their informed consent was obtained. A complete clinical history was then taken, and a physical examination was performed. If there were no symptoms or known diseases in the history and the examination was normal, the person was admitted to the study.

Electrodiagnostic studies were performed using an UltraPro S100 EMG/NCS/EP neurodiagnostic system (Natus Medical Incorporated). Surface self-adhesive electrodes were used to record motor and F responses. To obtain onset latency and baseline to peak amplitude, tibial nerve motor conduction studies were performed by stimulating the nerve trunk supramaximally at the ankle while recording the evoked CMAP from the abductor hallucis muscle. F waves were elicited at rest from the tibial nerve by supramaximal stimulation with a frequency of 1 Hz and 10–15 stimulations over the distal nerve and recording from the same muscle to obtain minimum, maximum, and mean latency and the difference between the maximum and minimum latency (chronodispersion) and the number of repetitions (persistence).

Acquisition conditions included a filter setting of 3 Hz to 10 kHz, a sweep speed of 8 ms per division and an amplifier gain of 0.2 millivolts per division for F waves and 5 mV for CMAP.

**Fig. 1:**
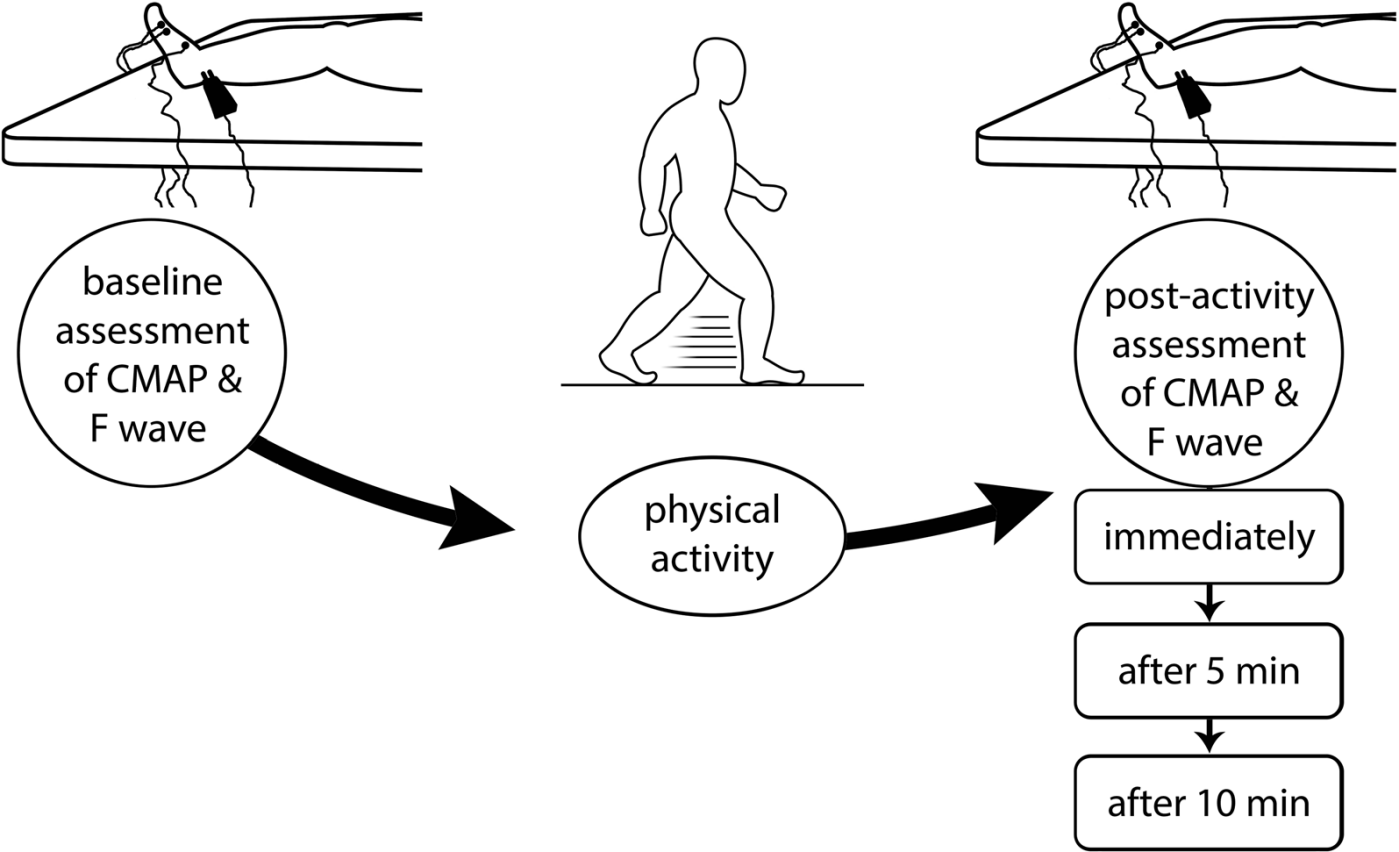
Schematic diagram of experiment methodology

Following baseline studies, patients were asked to walk at normal speed for 15 min [13, 17] without interruption in the clinic environment [18, 19]. The same electrophysiological parameters were recorded immediately after and in five and ten minutes upon completion of physical activity [12, 16] (Fig. 1).

### Ethical considerations

Before the project, all patients have been explained the objectives and methodology of the research and then declared informed consent. Following principles of ethics and confidentiality, all patient information remained confidential. This research was approved by Isfahan University of Medical Sciences, Iran (code of ethics: IR.MUI.MED.REC.1400.313).

### Statistical analysis

Data were analyzed using the Statistical Package for Social Sciences (SPSS) software version 21 using T-test, and repeated measures ANOVA. P-value < 0.05 was considered as the significance level.

## Results

**Fig. 2:**
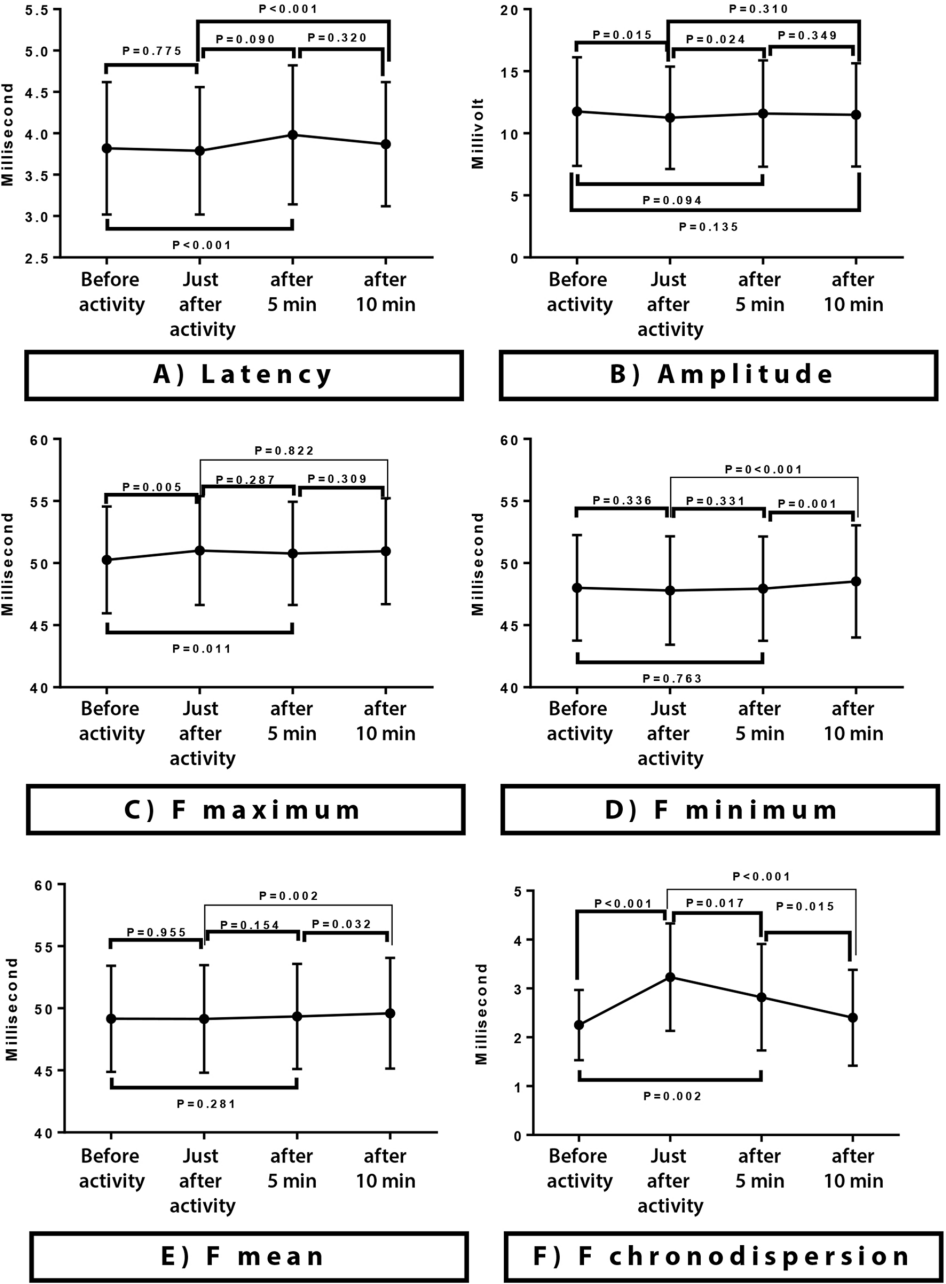
Pairwise comparison of tibial CMAP and F-wave parameters changes at different stages. A paired t-test is used to examine the stages in pairs. P values are reported according to pairwise comparisons. CMAP onset latency and amplitude **A** and **B**. Maximum F latency **C** shows incremental pattern just after activity and decremental pattern during rest. Minimum F latency **D** shows decremental pattern just after activity and incremental pattern during rest. Mean F latency **E** increases after activity. F chronodispersion **F**, increases significantly just after activity and decreases significantly with rest to approach pre-activity status

Based on the objectives of this study, CMAP latency and amplitude values, minimum latency, maximum latency, and mean latency of 10 F waves (F min, F max, and F mean, respectively), F chronodispersion, and F repetition percentage were evaluated and analyzed. A paired t-test (Fig. 2) was used to examine the groups in pairs. A repeated measures test (Table 1) was used to compare the intervention process before intervention to 10 min after intervention.

**Table 1:** Investigation of tibial CMAP and F wave parameters

	Before activity	After activity (immediately)	After activity (5 min)	After activity (10 min)
CMAP latency (ms)	3.82 ± 0.80	3.79 ± 0.77	3.98 ± 0.84	3.87 ± 0.75
CMAP amplitude (mV)	11.75 ± 4.38	11.25 ± 4.12	11.59 ± 4.28	11.49 ± 4.16
Minimum F-wave (ms)	48.00 ± 4.25	47.79 ± 4.39	47.94 ± 4.20	48.52 ± 4.52
Maximum F-wave (ms)	50.26 ± 4.30	51.01 ± 4.38	50.78 ± 4.16	50.96 ± 4.26
F-wave mean (ms)	49.03 ± 4.30	49.14 ± 4.40	49.30 ± 2.29	49.60 ± 4.49
F-wave chronodispersion (ms)	2.25 ± 0.72	3.23 ± 1.10	2.82 ± 1.09	2.40 ± 0.98
F-wave persistence (%)	99.12 ± 3.78	98.53 ± 5.57	98.24 ± 4.58	98.24 ± 5.20

Mean values of each parameter are reported in four phases (before and after activity) plus minus 2 standard deviations

*CMAP* compound muscle action potential, *ms* millisecond, *mv* millivolt

Immediately after the intervention, CMAP latency had almost the same values as before. It was not significantly different from before the intervention, but after 5 min, there was a significant upward trend compared to immediately after the intervention. CMAP amplitude also showed a significant decrease compared to before physical activity but gradually returned to its original level (pre-intervention values) over time (Table 1 and Fig. 2).

F min showed an insignificant decrease, whereas F max showed a significant increase after physical activity. Then, the trend of each parameter reversed to return to their baseline values. F min had an upward trend while F max had a downward trend, approaching their pre-intervention values. F chronodispersion, which actually results from the subtraction of the F min from the F max, reflected these trends. This means it showed a significant increase immediately after intervention and gradually reached the level before intervention with resting in the next stages. F means showed no significant changes after the intervention compared to before but F persistence showed significant changes comparing the same stages (Table 1 and Fig. 2).

## Discussion

F wave evaluation in different conditions [8] and after certain interventions [9–12] is a known technique for evaluating certain neurological pathologies, one of which is to record F waves before and after physical activity. Hence, basic normal values are considered a prerequisite for any Electrodiagnostic study, as this is the basis for conducting these studies in different populations [14, 15]. So in this study, dynamic F wave indices before and after physical activity in the lower extremities of healthy individuals were studied and analytically compared. Normal values of each index were determined and reported.

Manganotti et al. [13] reported that following exercise, F chronodispersion was the sole F wave parameter found to be significantly decreased for both peroneal and tibial nerves in their series of healthy subjects and suggested that this probably.

reflected a synchronization of motor neuron firing requiring a certain amount of descending facilitation. But our results showed a significant increase, not a decrease, in F chronodispersion after physical activity, which has the same trend observed in pathologic cases. In pathologies like spinal canal stenosis or radiculopathies F responses cannot be elicited right after exercise. Then F responses gradually emerge and show increased chronodispersion, which reduces during rest and approaches pre-exercise level [9]. Dynamic F wave parameters change in these pathologies is generally attributed to transient conduction block due to transient ischemia around nerve roots [9]. So it seems reasonable for healthy objects without underlying conditions inducing ischemia in the spinal canal to keep the same trend of chronodispersion changes with a milder slope, as it is discovered in our study.

Argyriou et al. [12] examined F wave changes in patients with peripheral arterial disease. They reported insignificant differences between pre-and post-exercise F wave values in the group of healthy controls.

Tsur et al. [11] conducted a study on the population of patients with lumbar radiculopathy. Results showed that following induction of fatigue with activity,

the dominant leg of healthy participants exhibited a significant reduction in nerve-muscle complex velocity (NMCV) and the relative CMAP amplitude. We also observed an insignificant reduction in CMAP amplitude post-activity.

Tsur et al. attributed differences in the results of various studies to the different experimental procedures: methods of causing fatigue, fatigue level, muscle properties, stimulation parameters, recording sites (mainly muscle? nerve?), etc.

Bal et al. [17] studied dynamic F wave in neurogenic intermittent claudication in lumbar spinal stenosis patients. However, they observed no significant difference between pre-and post-exercise F latency values of the healthy control group.

## Conclusion

Although physical activity showed statistically significant effects on some CMAP and F wave parameters (especially F chronodispersion), none of them exceeded normal clinical values introduced in the literature. So it is not expected for dynamic F wave parameters to differ significantly in healthy subjects concerning normal values. Therefore, changes beyond normal values in patients suspicious of certain diagnoses can be interpreted as abnormal along with other Electrodignostic tests and help to confirm the diagnosis.

## Limitations

This research is subject to several limitations. The sample size was sufficient for revealing statistical significance for some parameters, but insufficient for classification of results according to age groups and gender. The other limitation was unavailability the of the full text of the study reporting normal values of dynamic F wave by Manganotti et al. [13], which precluded us from further comparison and discussion of results.

## Data Availability

The datasets during and/or analysed during the current study available from the corresponding author on reasonable request.

## Abbreviations

CMAP: Compound muscle action potential
EP: Evoked potential
EMG: Electromyography
NCS: Nerve conduction study
NMCV: Nerve-muscle complex velocity
ms: Millisecond
mv: Millivolt
